# Pancreatic beta cell ER export in health and diabetes

**DOI:** 10.3389/fendo.2023.1155779

**Published:** 2023-04-21

**Authors:** Cesar Barrabi, Kezhong Zhang, Ming Liu, Xuequn Chen

**Affiliations:** ^1^ Department of Physiology, School of Medicine, Wayne State University, Detroit, MI, United States; ^2^ Center for Molecular Medicine and Genetics, School of Medicine, Wayne State University, Detroit, MI, United States; ^3^ Department of Endocrinology and Metabolism, Tianjin Medical University General Hospital, Tianjin, China

**Keywords:** pancreatic beta cells, endoplasmic reticulum (ER) export, coat protein complex II (COPII), proinsulin, diabetes

## Abstract

In the secretory pathway of the pancreatic beta cell, proinsulin and other secretory granule proteins are first produced in the endoplasmic reticulum (ER). Beta cell ER homeostasis is vital for normal beta cell functions and is maintained by the delicate balance between protein synthesis, folding, export and degradation. Disruption of ER homeostasis leads to beta cell death and diabetes. Among the four components to maintain ER homeostasis, the role of ER export in insulin biogenesis or beta cell survival was not well-understood. COPII (coat protein complex II) dependent transport is a conserved mechanism for most cargo proteins to exit ER and transport to Golgi apparatus. Emerging evidence began to reveal a critical role of COPII-dependent ER export in beta cells. In this review, we will first discuss the basic components of the COPII transport machinery, the regulation of cargo entry and COPII coat assembly in mammalian cells, and the general concept of receptor-mediated cargo sorting in COPII vesicles. On the basis of these general discussions, the current knowledge and recent developments specific to the beta cell COPII dependent ER export are summarized under normal and diabetic conditions.

## Introduction

The pancreatic beta cell is the only source to synthesize and secrete insulin. In the secretory pathway of the beta cells, proinsulin and other secretory as well as membrane proteins are first produced in the endoplasmic reticulum (ER), exported to the Golgi, and then packaged into insulin secretory granules (ISG) (1, 2). Beta cell ER possesses a highly active protein synthetic, folding, and export machinery to accommodate the massive production of proinsulin and other ISG proteins. ER homeostasis is vital for normal beta cell functions and is maintained by the delicate balance between protein synthesis, folding, export and degradation (1). Disruption of ER homeostasis by genetic and environmental diabetes-causing factors leads to beta cell dysfunction or death and subsequent diabetes (3, 4). Among the four components to maintain ER homeostasis, the role of ER export in insulin biogenesis and beta cell survival was not well-understood. From yeast to mammals, COPII (coat protein complex II) dependent transport is a conserved mechanism for most cargo proteins to exit ER and transport to Golgi apparatus (5–10). Emerging evidence revealed a critical role of COPII-dependent ER export in insulin biogenesis and beta cell survival (11–13). In this review, we will discuss the basic components of the COPII transport machinery, the regulations of cargo entry and COPII coat assembly, and the current knowledge of the COPII dependent ER to Golgi transport in beta cells under healthy and diabetic conditions.

## COPII dependent ER export in mammalian cells

### The Basic components of the COPII export machinery

**Figure 1:**
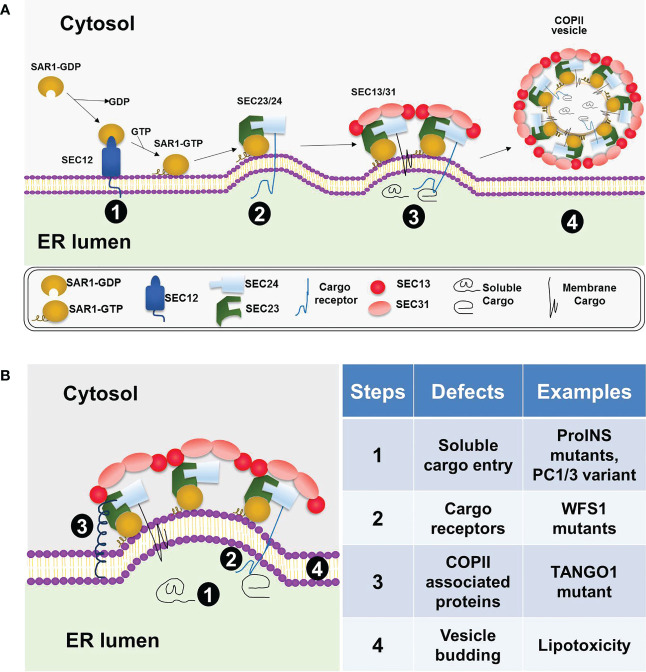
COPII coat assembly, cargo sorting, and vesicle formation at the ER. **(A)** A general model for COPII dependent ER exit in mammalian cells. COPII coat assembly on the ER membrane is initiated by SAR1 activation via SEC12 (stage 1), followed by the recruitment of the SEC23/SEC24 heterodimer and formation of the prebudding complex (stage 2). Next, the outer coat complex, SEC13/SEC31 heterotetramer, is recruited to bind the prebudding complex (stage 3). This step leads to further membrane deformation and vesicle formation. The vesicles coated with COPII proteins subsequently bud off the ER membrane (stage 4). **(B)** Defective ER export in beta cells under various diabetic conditions. Four different steps in the COPII-dependent ER exit process are highlighted on the left diagram and summarized with specific examples on the right table.

As shown in **Figure 1A**, according to the conventional model, the five COPII coat proteins, SAR1, SEC23, SEC24, SEC13 and SEC31, form a cage-like structure to sort cargo and bud off membrane vesicles, COPII vesicles, on specialized ribosome-free domains of the ER, known as ER exit sites (ERES) (6, 7, 14). Among them, the small GTPase, SAR1, initiates the coat assembly through GDP to GTP exchange mediated by its guanine nucleotide exchange factor SEC12. Upon activation, GTP bound SAR1 recruits SEC23.SEC24 heterodimer to form the inner coat and subsequently recruit SEC13.SEC31 heterotetramer to form the outer coat. Mammalian cells possess multiple isoforms of the COPII coat proteins, including SAR1A, SAR1B, SEC23A, SEC23B, SEC24A-D, SEC13, SEC31A, and SEC31B (6). Among the coat proteins, SEC24s directly interact and sort the cargo proteins into the COPII vesicles (15). Specific amino acid sequences are present in the cargo proteins which are recognized by the COPII coat. These sequences are called ER exit (or export) signals (or motifs) (16–18). The SEC24 isoforms have been shown to sort cargoes in an isoform- and tissue-specific manner (18–20). In addition to the five core coat proteins, other COPII associated proteins, such as SEC16, TANGO1/MIA3, and CTAGE5, also play essential roles in organizing the ERES and accommodating specific cargoes of large sizes (21–25). Several recent studies suggested that in addition to forming coated vesicles, COPII proteins can be recruited to membranes defining the boundary between the ER and ERES where they guide the cargo entry and extension of transport tubules without coating the ER export carriers (26, 27). While the basic machinery (5, 28) and structure of the COPII coat (29–32) have been well-understood, much remains to be learned regarding how COPII dependent cargo sorting and transport are regulated in a tissue-specific manner and how this regulated process may be disrupted in human diseases.

### The regulation of COPII-dependent ER export

According to current knowledge, COPII dependent cargo export is a highly regulated process (14, 33, 34). First, cargo entry into COPII vesicles can be regulated, primarily through allowing or inhibiting the interaction between cargo and coat component, SEC24. Such examples include SREBPs, a family of ER bound transcription factors whose COPII dependent ER-Golgi translocation and activation is regulated by cellular cholesterol levels through masking the ER exit signal (35, 36). Another example is ATF6, an ER stress sensor that translocates from ER to Golgi via COPII vesicles when activated under ER stress conditions (37, 38). CRTC2, a mediator of mTOR signaling activated by insulin, was shown to competes with SEC23A to interact with SEC31A, thus disrupting SREBP1 transport (39). It was also shown that the ER/lipid droplet-associated protein Cideb selectively promoted the loading of SREBP/SCAP into COPII vesicles (40). These examples support the promise to design drugs targeting the cargo specific COPII isoforms or the interaction interfaces between specific cargo and COPII coat. The second aspect of regulation is at the COPII coat assembly on ERES. This process can be modulated by various factors such as cargo load (41–43), metabolic conditions (44, 45), or membrane lipids (46, 47). This level of regulation can be achieved through multiple mechanisms targeting different steps of coat complex assembly. For example, such regulation can be achieved at the gene expression level. It has been shown that COPII proteins were targets of unfolded protein response (UPR), and the IRE1α-mediated UPR branch regulated the expression of genes involved in ER-Golgi transport (41, 45, 48). The COPII-dependent ER export can also be regulated by post-translational modifications of the COPII coat and related proteins (33, 34). Examples of such modifications include the ubiquitylation of SEC31 (49, 50), phosphorylation of SEC24 (51), SEC16 (52), SEC12 (53), and O-GlcNAcylation of multiple COPII components (54, 55). In mammalian cells, in response to nutrient starvation, COPII proteins can be redirected to the autophagosome formation through phosphorylating SEC23B subunit (56). Interestingly, COPII-dependent ER protein transport can also be regulated by the circadian clock (57). The ER-bound stress sensor CREBH (CAMP-Responsive Element-Binding Protein, Hepatic-Specific) transits from the ER to Golgi via COPII vesicle for its activation process in response to hepatic stress, nutrient availability, or circadian cues (57–59). Under the physiological condition, the interaction between CREBH and SEC23/SEC24 and the subsequent proteolytic activation of CREBH exhibited typical circadian rhythmicity. This circadian-regulated process was controlled by the core clock oscillator BMAL1 and AKT/glycogen synthase kinase 3β (GSK3β) signaling cascade, in which the GSK3β-mediated phosphorylation of CREBH modulated the association between CREBH and SEC23/SEC24 (57). In addition to transcriptional and post-translational regulations described above, modulation of SAR1 GTPase activity has also been shown as another mechanism to regulate the COPII-dependent ER export (60, 61).

### Receptor mediated cargo sorting into COPII vesicles

Soluble secretory proteins locate in the ER lumen and therefore cannot interact with cytoplasm-sided COPII coat proteins directly. These protein cargos enter the COPII vesicles either via bulk flow or via selective concentration through interacting with specific ER membrane proteins, called cargo receptors (8, 62). By bridging the interaction between cargos in the ER lumen and COPII proteins on the cytoplasmic side of the ER membrane, cargo receptors mediate the efficient ER export of selective proteins (63). In addition to soluble cargos, some membrane cargos have conformational constraints that prevent their direct interactions with the COPII coat. Therefore, these proteins also need cargo receptors to facilitate their entry into the COPII vesicles. After incorporation into COPII transport vesicles, cargo receptors release bound cargo in pre-Golgi or Golgi compartments, and then recycled back to the ER for additional rounds of cargo export (62). Different types of cargo receptors that recognize carbohydrate and/or polypeptide signals in secretory cargos have been characterized. Some of most extensively studied mammalian cargo receptors included LMAN1 (also known as ERGIC-53) (64) and SURF4 (65). Loss-of-function mutation in LMAN1 causes combined factor V and factor VIII deficiency in human (66) and Lman1 deficient mice exhibited reduced plasma levels of factor V and factor VIII as well as ER accumulation of α1-antitrypsin in hepatocytes (67). As the mammalian homolog of the yeast COPII cargo receptor Erv29p, SURF4 has long been hypothesized as a cargo receptor in mammalian cells. However, its putative cargos have only been identified recently and were discussed extensively in two recent well-written reviews (63, 68). Despite affecting a broad range of cargos in cell culture, SURF4 exhibited, in several recent *in vivo* studies, a striking priority for lipoproteins in the liver. SURF4 appears to play an important role in ER export of lipoproteins and therefore regulating lipid homeostasis *in vivo* (69–71).

## COPII dependent ER export in pancreatic beta cell

### COPII dependent ER export and insulin biogenesis

Proinsulin is the major soluble cargo in beta cells. Its biosynthesis alone can account for up to 50% of the total protein synthesis under glucose-stimulated conditions (72, 73). However, the molecular mechanism of proinsulin ER export was poorly understood until recent years. To understand the role of the COPII machinery in proinsulin ER export and insulin biogenesis, dominant negative SAR1 mutants were over-expressed to specifically block COPII-dependent ER export in MIN6 cells (11), isolated mouse and human islets (11, 74). Results from these studies demonstrated that SAR1 mutants blocked the export of mCherry-tagged as well as the endogenous proinsulin from exiting the ER and abolished the conversion of proinsulin to insulin. These effects were confirmed by siRNA-based Sar1A and Sar1B double knockdown experiments in MIN6 cells (11, 74). Furthermore, when a well-established *in vitro* COPII budding assay was applied to MIN6 cells, proinsulin was shown to be incorporated into COPII vesicles with the same ATP, GTP, and SAR1 dependence as other well-characterized COPII cargo proteins including Lman1 and Sec22B (11). Collectively, results from these studies firmly established that the COPII-dependent transport machinery is required for proinsulin ER export and insulin biogenesis (11, 74).

### Knockout mouse model for beta cell ER export

Consistent with the key findings in the beta cell line and isolated islets (11, 74), mice with beta cell–specific knockout of Ctage5, a key player of COPII dependent ER export, also showed impaired proinsulin trafficking and reduced insulin biogenesis (12). In pancreatic sections of the wild type mice, proinsulin immunofluorescent signals were detected throughout the ER, ERGIC (ER-Golgi intermediate compartment) and Golgi, whereas they were primarily in the ER in the conditional knockout mice (12). This finding is consistent with an impaired proinsulin ER export. A similar result was found in the Sar1A/1B double knockdown MIN6 cells (74). In the knockdown cells, the impaired COPII vesicle formation resulted in ER retention of proinsulin as evidenced by the increased colocalization of proinsulin with the ER marker, protein disulfide isomerase (74). In the Ctage5 knockout mice study, it was further revealed that Ctage5 interacted with vesicle SNARE protein, Sec22b, and may coordinate with Sec22b to control the release of COPII vesicles from the ER (12). It is interesting to note that a human TANGO1 mutation was recently identified to cause insulin-dependent diabetes mellitus along with skeleton defects related to impaired collagen secretion (75). Since the diabetes is due to reduced plasma insulin (75) and TANGO1 is known to interact and cooperate with CTAGE5 (24), further investigation is warranted to understand the role of TANGO1 in beta cell and insulin secretion.

## Potential cargo receptors for ER export of proinsulin and other secretory proteins in beta cells

The above discussed studies revealed that the ER export of proinsulin was COPII-dependent and efficient ER export was essential for beta cell functions (11, 12, 74). However, the molecular mechanism by which proinsulin and other secretory cargos are packaged into COPII vesicles was not clearly understood. Particularly, it was unknown until recently whether their ER export is mediated by membrane cargo receptor(s). Interestingly, a recent study showed that WFS1 acted as a cargo receptor for beta cell ER export of proinsulin as well as other secretory proteins (13). In this study, an abnormal ER accumulation of proinsulin immunofluorescent signals was observed in Wfs1 knockdown but not the control INS-1 cells. This finding was confirmed in the whole-body Wfs1 knockout mice where proinsulin was mainly colocalized with the ER markers in the beta cells (13). These results together indicated that Wfs1 is required for ER export of secretory cargo proteins such as proinsulin in beta cells. Consequently, Wfs1 knockdown or knockout led to a significantly increased proinsulin to insulin ratio (13).

Consistent with being a cargo receptor for soluble cargoes, Wfs1 interacted with Sec24 isoforms via its cytosolic N-terminal domain which contains two consensus di-acidic ER export signals, ^158^ENE and ^169^ETD (13). Mutagenesis of either signal abolished the interaction between Wfs1 and Sec24. Using Bimolecular Fluorescence Complementation assay and Proximity Ligation Assay in HEK-293T cells and endogenous cargo protein immunoprecipitations in INS-1 cells, it was further shown that Wfs1 could directly interact, through its luminal C-terminal domain, with proinsulin as well as other vesicular cargo proteins such as Cpe and Scg5 (13). Interestingly, several pathogenic WFS1 mutations in Wolfram Syndrome are located within these two critical interacting domains. While the N-terminal WFS1 mutants disrupted the interaction between Wfs1 and Sec24, the C-terminal mutants failed to interact and recognize the cargo proteins (13).

In another recent study, Surf4 was shown to regulate the ER export of proinsulin in INS-1 832/13 cells (76). Under high-glucose condition, Surf4 expression was upregulated and predominantly localized to the ERES. Surf4 knockdown resulted in proinsulin retention in the ER and decreased level of mature insulin in the secretory granules. Furthermore, proinsulin could be coimmunoprecipitated with Surf4 when both proteins were overexpressed in INS-1 832/13 cells. These results supported Surf4 as a cargo receptor for proinsulin ER export in beta cell line (76). Recently, liver-specific knockout mice studies have shown that Surf4 played an important role, as a cargo receptor, in ER export of lipoproteins *in vivo* (69–71). In future studies, it will be interesting to examine the *in vivo* function of Surf4 in a beta cell specific Surf4 knockout mouse model.

## Defective ER export in beta cell dysfunction and diabetes

ER homeostasis is vital for normal beta cell functions and is maintained by the delicate balance between protein synthesis, folding, export and degradation. Disruption of this balance by genetic and environmental diabetes-causing factors leads to ER stress, beta cell death and diabetes (4, 73). Defective UPR signaling can disrupt ER homeostasis and normal beta cell functions (77). For example, defect in the UPR transducer PERK or IRE1α function led to poor beta cell survival (78–80). Mutations in the PERK gene cause a human diabetic condition known as Wolcott-Rallison syndrome, a disease state that can be observed in PERK knockout mice (78, 81). Recent studies in MIN6 cells, isolated islets, and beta cell specific Ctage5 KO mice showed that impaired COPII-dependent ER export strongly disrupted ER homeostasis, induced ER stress, and resulted in beta cell apoptosis (11, 12). Furthermore, the ER stress induced by defective ER export was mediated via the PERK/pEIF2α and IRE1/Xbp1 pathways but not via ATF6 because its own activation was also inhibited under this condition (11). These results in beta cell line and animal model showed that defective ER export could contribute to beta cell ER stress and dysfunction. It will be important to determine the association between beta cell dysfunction in various forms of human diabetes and mutations of the COPII or the associated proteins. In this regard, the recent finding of a human TANGO1 mutation to cause insulin-dependent diabetes mellitus (75) is promising and warrant further investigation.

A follow-up study of the dominant negative SAR1 mutants in MIN6 cells and isolated islets linked defective ER export to impaired proinsulin oxidative folding in beta cell ER (74). Under this condition, misfolded proinsulin formed aberrant disulfide-linked dimers and high molecular weight proinsulin complexes. Since proinsulin is the most abundant protein in the ER and its synthesis alone accounts for up to 30-50% of total protein synthesis of beta cells stimulated by high glucose, increased misfolded proinsulin is likely an important and direct contributor to ER stress induced by defective ER export. This argument is supported by alleviation of ER stress through limiting proinsulin load in the ER using Ins1 and Ins2 knockdown (74). This study revealed the significance of efficient ER export in maintaining ER protein homeostasis and native folding of proinsulin. Given the fact that proinsulin misfolding plays an important role in diabetes (1, 73, 82–84), this study suggested that enhancing ER export may be a potential therapeutic target to prevent/delay beta-cell failure caused by proinsulin misfolding and ER stress. Additional approaches that may lessen the ER stress and beta cell failure caused by misfolded proinsulin include promoting ER associated degradation (ERAD) of proinsulin (85) and/or decreasing disulfide-linked misfolded proinsulin complexes by limiting formation of abnormal intermolecular disulfide bonds (86).

In human studies, emerging genetic evidences and functional studies in human beta cell models linked defective ER-Golgi transport to insulin deficiency and diabetes. As already discussed, a human TANGO1 truncating mutation was recently identified to cause insulin-dependent diabetes (75). In another recent human study, homozygous mutations in the YIPF5 gene were found to cause neonatal diabetes (87). YIPF5 is a multi-spanning membrane protein localized in the ER, ERGIC, as well as Golgi apparatus and plays an important role in trafficking between the ER and Golgi (88–91). Deficiency of YIPF5 in human pancreatic beta-cell models, including YIPF5 silencing in EndoC-betaH1 cells, YIPF5 knockout and mutation knockin in ESCs, and patient derived iPSCs, were used to investigate the underlying mechanisms (87). Loss of YIPF5 function in stem cell–derived islet cells resulted in proinsulin retention in the ER, marked ER stress, and beta cell failure (87). The beta cell defects found in the YIPF5 knockout are quite similar to the phenotype caused by disrupting Wfs1 (see Section above). It would be interesting to examine whether YIPF5 is required for selective cargo sorting, especially for the soluble cargoes, into COPII vesicles. While there are evidences supporting YIPF5’s role in ER membrane organization and cargo exit at ERES (88, 90, 92), there is also evidence to show that YIPF5 regulates retrograde transport from Golgi to the ER (93). Therefore, it will be worthwhile to determine the primary function of YIPF5 in beta cell ER-Golgi transport in future studies. While the above studies showed that defective ER export machinery can lead to beta cell dysfunction and death, deficiencies of individual cargo proteins in their ER export capabilities have also been linked to disease conditions. It was found that some mutations in ATP-sensitive potassium channel caused human congenital hyperinsulinism and one of these mutations disrupted the ER exit signal on the Kir6.2 channel (94). GWAS study showed prohormone convertase 1/3 (PC1/3) variants were associated with fasting proinsulin levels (95), and defective PC1/3 ER export could potentially contribute to impaired insulin biogenesis in diabetes (96).

Evidences also exist that environmental diabetogenic factors, particularly lipotoxicity, reduced ER-Golgi transport and induced ER stress in beta cells (97–99). Using GFP-tagged, temperature-sensitive vesicular stomatitis virus G protein (VSVG) to quantify the rate of ER-to-Golgi protein trafficking, it was found that pretreatment with palmitate, to mimic lipotoxic condition *in vitro*, significantly reduced the rate of ER-Golgi protein transport in MIN6 cells (97, 100). Using the same reporter, high-density lipoproteins were found in a later study to restore ER-Golgi trafficking in palmitate-treated MIN6 cells (101). As for the potential molecular mechanisms by which saturated fatty acids impaired ER export, a subsequent study found that chronic palmitate treatment disrupted ER lipid rafts and inhibited vesicle budding from the ER (98). Using the same temperature-sensitive VSVG-GFP reporter, another diabetes contributing factor, hypoxia, was shown to reduce ER-Golgi protein trafficking in MIN6 cells (102). These findings suggest that defective COPII dependent cargo export could be one of the underlying causes for the major beta cell defects in diabetes including impaired proinsulin maturation, loss of insulin content, abnormal insulin granule morphology, chronic ER stress and beta cell apoptosis in diabetes (1, 4, 73, 103). In fact, defective insulin maturation with increased ER localization of proinsulin was reported in the beta cells of type 2 diabetes patients (104). It will be of importance to determine if ERES organization or COPII coat assembly is altered in human diabetic islets. It is worth noting that under other circumstances, defective COPII dependent cargo export may be a consequence of chronic beta cell ER stress and dysfunction in diabetes rather than its primary cause.

## Future perspective

Building on the well characterized COPII machinery and recent studies of COPII dependent ER export in beta cells, the essential roles of this pathway in proinsulin trafficking, insulin biogenesis and beta cell ER homeostasis have been established. Next questions to be answered will be the physiological regulation of this process in health and its dysregulation under various diabetic conditions. For example, how does the COPII-dependent ER export pathway adapt to the fluctuations of cargo (proinsulin etc.) outflow between low and high glucose? How does it prevent improperly folded cargo molecules from entering the COPII vesicles and what roles do the identified cargo receptors play in this regard? How diabetogenic factors, lipotoxicity or cytokines etc., may disrupt this process? How is ERES organization, COPII coat assembly, or COPII protein expression/PTMs altered in human diabetic islets and to what extent these changes can contribute to the defective insulin biogenesis? As we are just at the beginning of understanding these important issues, more exciting and novel scientific findings are expected and new therapeutic opportunities may unveil in the near future.

## Author contributions

All authors listed have made a substantial, direct, and intellectual contribution to the work and approved it for publication.
